# Supplementary material for: Novel Driver Strength Index highlights important cancer genes in TCGA PanCanAtlas patients
Source: PeerJ. 2022 Aug 11;10:e13860. doi: 10.7717/peerj.13860 (PMC9375969; doi:10.7717/peerj.13860)
Supplement: Supplemental Information 2 [file peerj-10-13860-s002.zip › COHORTS/genes plots/age/2021_8_16_14_9_distribution_cohorts_70-74.pdf]

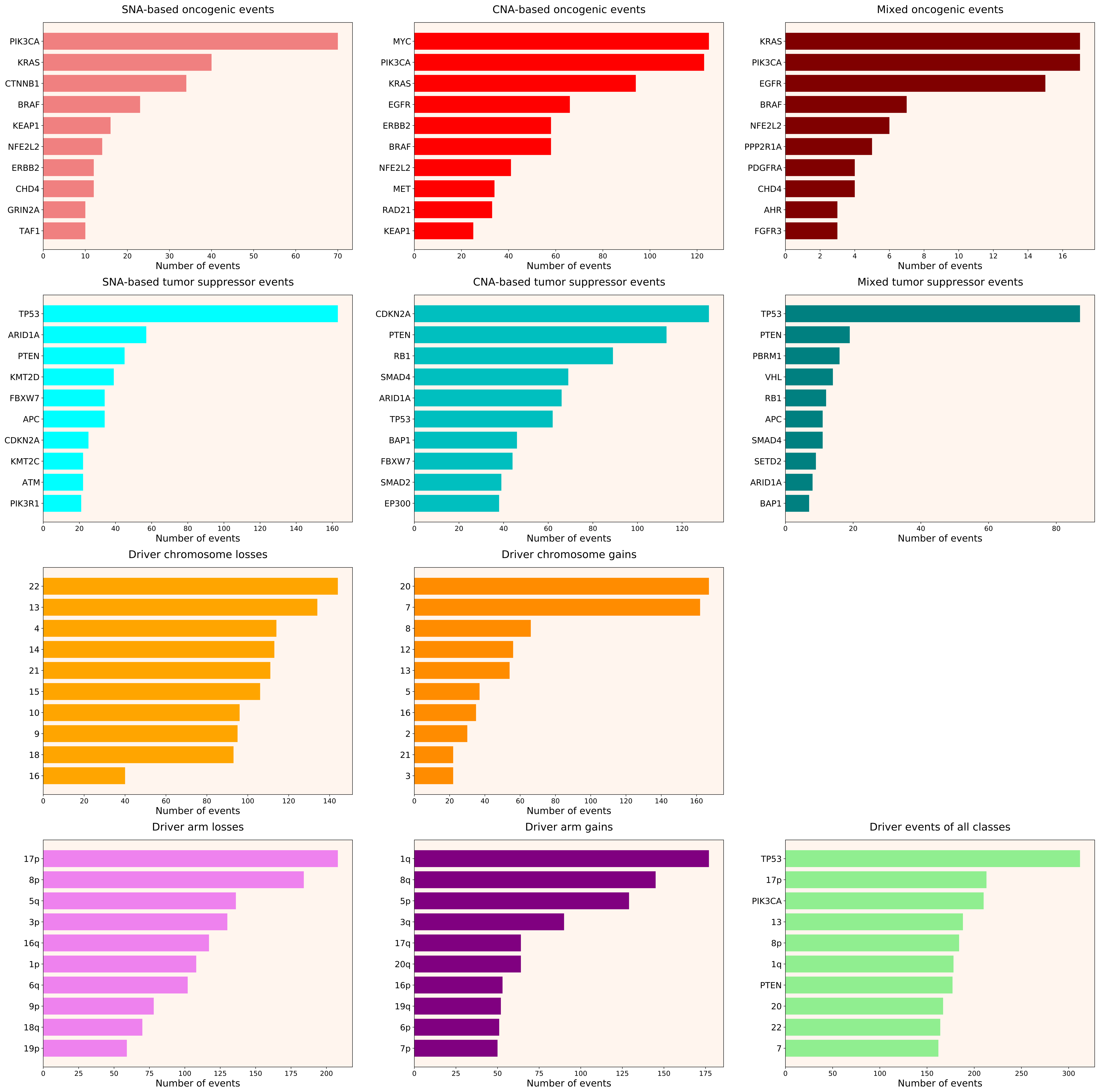

SNA-based oncogenic events
PIK3CA
KRAS
CTNNB1
BRAF
KEAP1
NFE2L2
ERBB2
CHD4
GRIN2A
TAF1
0 10 20 30 40 50 60 70
Number of events
CNA-based oncogenic events
MYC
PIK3CA
KRAS
EGFR
ERBB2
BRAF
NFE2L2
MET
RAD21
KEAP1
0 20 40 60 80 100 120
Number of events
Mixed oncogenic events
KRAS
PIK3CA
EGFR
BRAF
NFE2L2
PPP2R1A
PDGFRA
CHD4
AHR
FGFR3
0 2 4 6 8 10 12 14 16
Number of events
SNA-based tumor suppressor events
TP53
ARID1A
PTEN
KMT2D
FBXW7
APC
CDKN2A
KMT2C
ATM
PIK3R1
0 20 40 60 80 100 120 140 160
Number of events
CNA-based tumor suppressor events
CDKN2A
PTEN
RB1
SMAD4
ARID1A
TP53
BAP1
FBXW7
SMAD2
EP300
0 20 40 60 80 100 120
Number of events
Mixed tumor suppressor events
TP53
PTEN
PBRM1
VHL
RB1
APC
SMAD4
SETD2
ARID1A
BAP1
0 20 40 60 80
Number of events
Driver chromosome losses
22
13
4
14
21
15
10
9
18
16
0 20 40 60 80 100 120 140
Number of events
Driver chromosome gains
20
7
8
12
13
5
16
2
21
3
0 20 40 60 80 100 120 140 160
Number of events
Driver arm losses
17p
8p
5q
3p
16q
1p
6q
9p
18q
19p
0 25 50 75 100 125 150 175 200
Number of events
Driver arm gains
1q
8q
5p
3q
17q
20q
16p
19q
6p
7p
0 25 50 75 100 125 150 175
Number of events
Driver events of all classes
TP53
17p
PIK3CA
13
8p
1q
PTEN
20
22
7
0 50 100 150 200 250 300
Number of events
